# Polydeoxyribonucleotide (PDRN) in Dentistry: Narrative Review for Mechanisms and Emerging Clinical Applications

**DOI:** 10.1007/s13770-025-00776-z

**Published:** 2026-01-17

**Authors:** Jeong-Kui Ku, Pil-Young Yun, Yeong Kon Jeong

**Affiliations:** 1 Department of Oral and Maxillofacial Surgery, Section of Dentistry, Seoul National University Bundang Hospital, 300 Gumi-dong, Bundang-gu, Seongnam, Gyeonggi-do 13620 Republic of Korea; 2 Department of Dentistry and Dental Research Institute, School of Dentistry, Seoul National University, Seoul, 03080 Republic of Korea; 3 Department of Oral and Maxillofacial Surgery, College of Dentistry, Wonkwang University, Iksan, Korea

**Keywords:** Polydeoxyribonucleotide (PDRN), Angiogenesis, Osteogenesis, Regenerative Dentistry, Mechanism

## Abstract

**Background:**

Polydeoxyribonucleotide (PDRN) has emerged as a promising and cost-effective biological agent in regenerative medicine due to its anti-inflammatory, angiogenic, and tissue-regenerative properties.

**Methods:**

This review outlines the mechanisms of action of PDRN, namely activation of the A_2_A receptor and nucleotide provision via the salvage pathway, and summarizes its biological roles in dental regeneration together with current preclinical and clinical evidence.

**Results:**

In dentistry, PDRN has been shown to enhance osteogenesis and vascularization when used with bone graft scaffolds, to exert anti-inflammatory and chondroprotective effects in temporomandibular joint disorders, and to modulate pain pathways in neuropathic conditions. It has also demonstrated adjunctive benefits in managing inflammatory oral diseases such as peri-implantitis and medication-related osteonecrosis of the jaw, where its dual regenerative and anti-inflammatory actions support both soft- and hard-tissue healing.

**Conclusion:**

Although these findings highlight broad therapeutic potential, current evidence remains limited. Most reports derive from preclinical experiments or small-scale clinical studies, and well-designed randomized controlled trials are needed to validate efficacy of PDRN and define its optimal clinical indications in evidence-based dental protocols.

## Introduction

Polydeoxyribonucleotide (PDRN) is a DNA-derived compound primarily extracted from the sperm of *Oncorhynchus mykiss* (Salmon Trout) or *Oncorhynchus keta* (Chum Salmon), consisting of polymeric chains of deoxyribonucleotides with molecular weights ranging from 50 to 1500 kDa [1]. From a single salmon or trout, approximately 5–10 mL of milt (sperm) can be obtained to yield these low-molecular-weight DNA fragments, including PDRN and other polynucleotides.^2^ In practical terms, PDRN is essentially a mixture of DNA fragments. By convention, the term “PDRN” refers to short- and medium-length deoxyribonucleotide chains (molecular weight < 1500 kDa), whereas “PN” (polynucleotide) denotes longer chains (≥ 1500 kDa) [2]. PDRN is not metabolized by the liver; instead, it is broken down by non-specific DNases (in plasma or bound to cell membranes) and its metabolites are excreted in the urine. The biological activity of PDRN is largely attributed to the generation of adenosine, a nucleoside composed of a deoxyribose sugar and adenine (a purine base) [3].

In the 1990s, several studies demonstrated PDRN’s efficacy in stimulating fibroblast proliferation, drawing attention to its potential application in wound healing [4, 5]. In 1994, the first drug containing PDRN as an active principle (Placentex®; Mastelli, Sanremo, Italy) was launched, originally approved as an injectable treatment for dystrophic or dystrophic–ulcerative connective tissue disorders (essentially to prevent pathologic scar formation as an anti-dystrophic agent) [6]. PDRN exerts its biological effects via two principal mechanisms: activation of the adenosine A_2__A_ receptor and the provision of deoxyribonucleotide substrates through the salvage pathway [7]. These properties have been extensively investigated in various medical fields such as dermatology, orthopedics, and plastic surgery, where PDRN has shown clinical efficacy in the treatment of diabetic ulcers, pressure sores, tendon injuries, and ischemic wounds [7–10].

In recent years, the scope of PDRN has expanded into dental medicine. Oral tissues are frequently subjected to trauma, infection, and surgical interventions, all of which demand rapid healing and tissue remodeling. PDRN’s unique biological characteristics—including promotion of extracellular matrix remodeling, stimulation of angiogenesis, and modulation of inflammatory cytokines—make it a promising agent for dental applications such as periodontal regeneration, bone grafting, implantology, soft tissue augmentation, and the treatment of medication-related osteonecrosis of the jaw (MRONJ) [11–15]. This review aims to describe the mechanisms of PDRN and provide a comprehensive overview of the current evidence supporting its applications in dental research and clinical practice.

## Biological mechanisms of PDRN

**Fig. 1:**
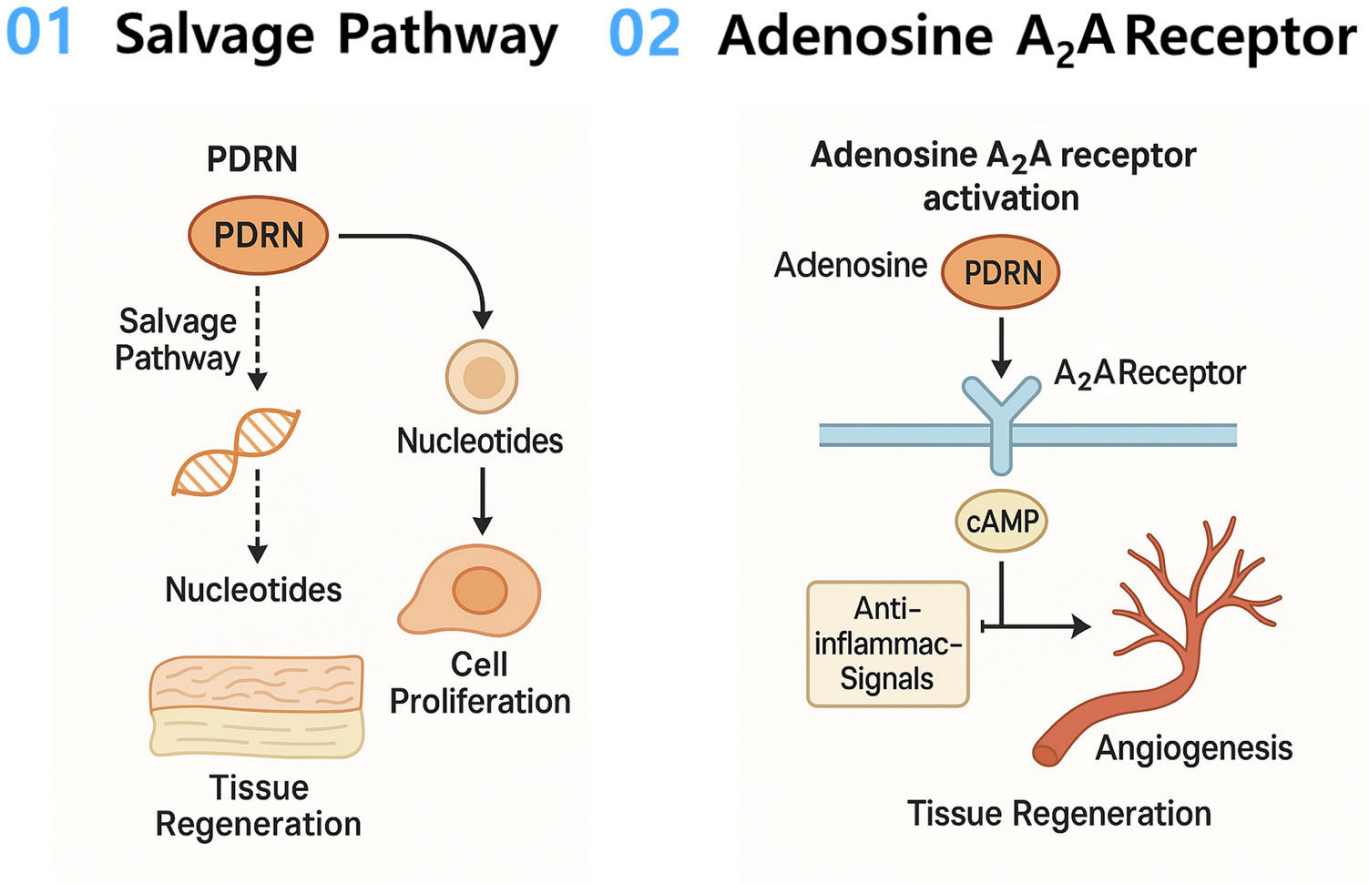
Schematic diagram of the mechanisms of PDRN in tissue regeneration*.* PDRN acts via two main pathways: (1) Salvage pathway activation, whereby PDRN is broken down into nucleotides that are recycled for DNA/RNA synthesis in repairing cells. This conserves cellular energy by reutilizing nucleotide building blocks, accelerating DNA repair, cell proliferation, collagen synthesis, and bone formation. (2) A_2_A receptor activation, which increases cAMP and triggers signaling cascades that suppress inflammation and promote angiogenesis.

PDRN’s therapeutic effects in tissue regeneration are primarily achieved through two mechanisms: (1) the salvage pathway, and (2) activation of adenosine A_2__A_ receptors (Fig. 1). First, through the salvage pathway, PDRN’s degradation products serve as a source of nucleotides for cells, promoting DNA synthesis and cell proliferation in damaged tissues. In other words, by recycling nucleotides—the building blocks of nucleic acids (DNA and RNA)—cells conserve the energy and resources that would be required to synthesize new nucleotides de novo [16]. Adenosine, a nucleoside originating from PDRN breakdown, interacts with purinergic P2X and P2Y receptors (P2XR and P2YR), adenosine A_2__A_ and A_2__B_ receptors, and nicotinic acetylcholine receptors (nAChR) to regulate cellular and tissue functions, thereby stimulating pathways for proliferation, differentiation, maturation, and anti-inflammation [16]. Moreover, PDRN has been shown to modulate key signaling cascades in a mechanism analogous to adenosine. It suppresses the nuclear factor kappa B (NF-κB) pathway and activates the Wnt/β-catenin pathway, modulating the inflammatory response and playing an immunomodulatory role in tissue regeneration [17].

### Salvage pathway and nucleotide supply

In parallel with receptor-mediated effects, PDRN contributes to tissue regeneration through the “salvage pathway” of nucleotide synthesis [1]. PDRN is a mixture of double-stranded DNA fragments approximately 80–2200 base pairs in length, which are depolymerized by endogenous DNases into free purine and pyrimidine nucleosides and bases [18]. These liberated bases are then reutilized by cells to synthesize new DNA and RNA without expending the energy required for de novo nucleotide synthesis [9, 18]. PDRN’s nucleotide supply via the salvage pathway is particularly important under conditions of cellular stress or injury (e.g., hypoxia or trauma), when rapid cell proliferation is needed for healing. By using this energy-efficient salvage route instead of the ATP-intensive de novo pathway, cells in damaged or ischemic tissues can more readily achieve DNA repair and replication, thus accelerating tissue repair. PDRN breakdown products effectively support the replication of fibroblasts, osteoblasts, and other reparative cells, enhancing tissue turnover and wound healing. For example, providing exogenous deoxyribonucleotides has been shown to mitigate DNA damage and promote cell survival in ischemic or UV-damaged tissues [8, 9, 18, 19]. This salvage pathway is less energy-consuming and more efficient under conditions of cellular stress, which are commonly observed in inflamed or surgically injured oral tissues.

### Adenosine A_2_A receptor activation

PDRN is also known to act as a selective agonist of the adenosine A_2_A receptor, a G protein–coupled receptor widely expressed on immune cells, fibroblasts, and endothelial cells [1]. As PDRN is broken down by nucleases, the resulting adenosine molecules can bind to A_2_A receptors on cells such as fibroblasts [2, 20]. A_2_A receptor engagement leads to an increase in intracellular cyclic AMP (cAMP) and activates downstream signaling cascades that orchestrate an anti-inflammatory and pro-regenerative response. Specifically, A_2_A activation by PDRN suppresses the production of pro-inflammatory cytokines (e.g., tumor necrosis factor-α, interleukin-6) while increasing anti-inflammatory signals [21]. Concurrently, PDRN’s agonism of A_2_A receptors upregulates factors like vascular endothelial growth factor (VEGF), angiopoietin-1, and matrix metalloproteinase-2, all of which facilitate neovascularization in healing tissues [11]. This receptor-mediated mechanism enhances new blood vessel formation and modulates the inflammatory milieu, creating a more favorable environment for tissue healing [12]. Moreover, PDRN’s activation of A_2_A receptors has been shown to reduce NF-κB pathway activation and apoptosis in injured cells, thereby accelerating the resolution of inflammation. It downregulates key inflammatory mediators including IL-1β, TNF-α, inducible nitric oxide synthase (iNOS), and IL-6, while promoting tissue survival and repair [20, 22]. Importantly, the regenerative benefits of PDRN are abolished by A_2_A antagonists such as 3,7-dimethyl-1-propargylxanthine (DMPX), confirming that A_2_A receptor signaling is a primary pathway for its activity [18].

**Fig. 2:**
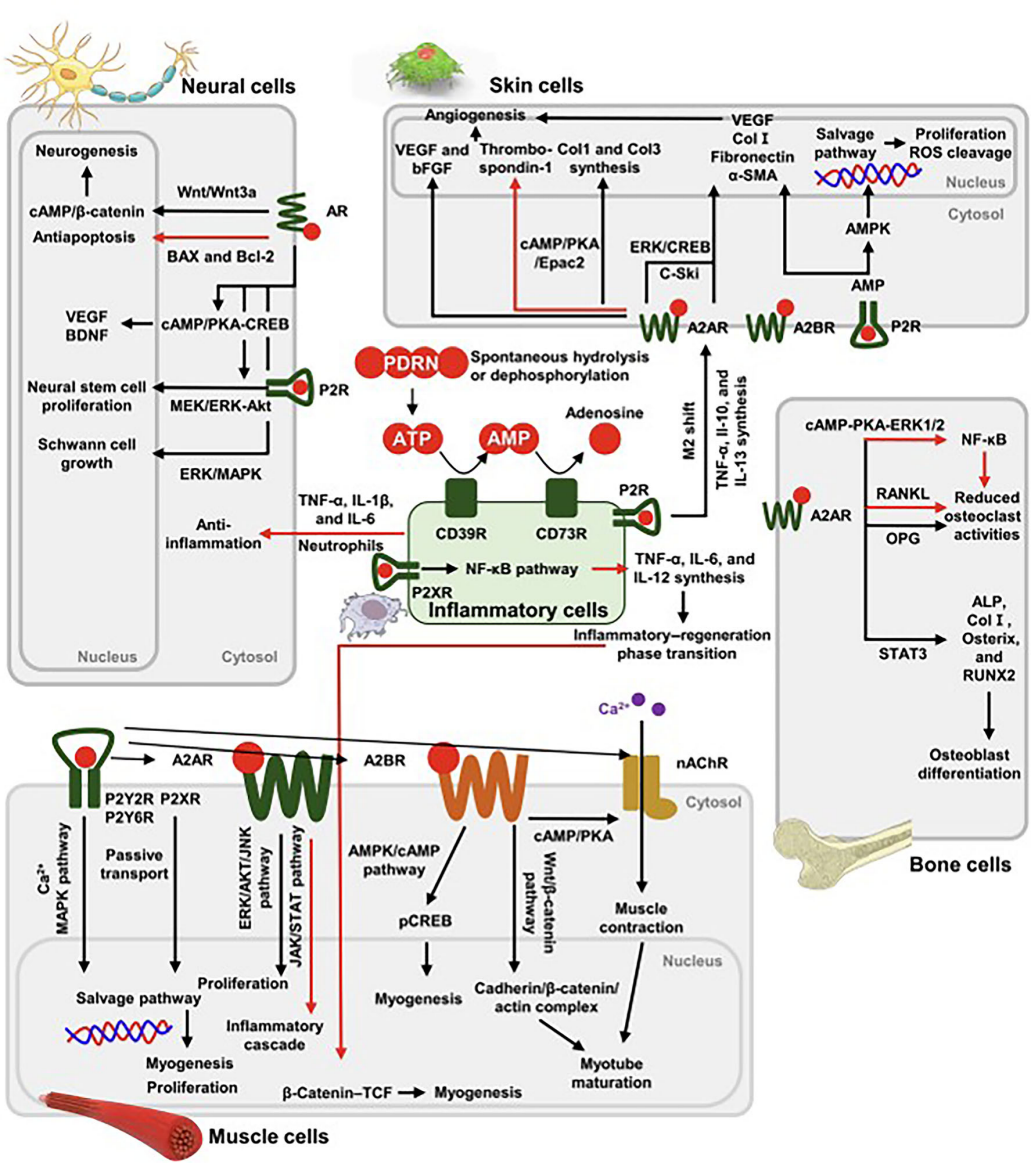
Schematic summary of the representative signal pathways of polydeoxyribonucleotide (PDRN) for tissue regeneration. (Adapted from Oh et al., *Biomaterials Research*, 2025, CC BY 4.0 [23]) (A2AR, adenosine A2A receptor; A2BR, adenosine A2B receptor; ALP, alkaline phosphatase; α-SMA, α-smooth muscle actin; AMPK, AMP-activated protein kinase; AMP, adenosine monophosphate; AR, androgen receptor; ATP, adenosine triphosphate; BAX, Bcl-2-associated X protein; Bcl-2, B-cell lymphoma 2; BDNF, brain-derived neurotrophic factor; bFGF, basic fibroblast growth factor; cAMP, cyclic adenosine monophosphate; Col I, type I collagen; Col3, type III collagen; CREB, cAMP response element-binding protein; Epac2, exchange protein directly activated by cAMP 2; ERK, extracellular signal-regulated kinase; IL-1β, interleukin-1 beta; IL-6, interleukin-6; IL-10, interleukin-10; IL-12, interleukin-12; IL-13, interleukin-13; JAK, Janus kinase; JNK, c-Jun N-terminal kinase; MAPK, mitogen-activated protein kinase; MEK, mitogen-activated protein kinase kinase; nAChR, nicotinic acetylcholine receptor; NF-κB, nuclear factor kappa B; OPG, osteoprotegerin; P2R, purinergic receptor; P2XR, purinergic P2X receptor; P2Y2R, purinergic P2Y2 receptor; P2Y6R, purinergic P2Y6 receptor; pCREB, phosphorylated CREB; PKA, protein kinase A; RANKL, receptor activator of nuclear factor κB ligand; ROS, reactive oxygen species; RUNX2, Runt-related transcription factor 2; STAT3, signal transducer and activator of transcription 3; TCF, T-cell factor; TNF-α, tumor necrosis factor-alpha; VEGF, vascular endothelial growth factor)

Notably, this dual mode of action—A_2A_ receptor activation coupled with salvage pathway nucleotide provision—may confer PDRN a unique advantage over single-pathway agents. PDRN not only initiates pro-healing signaling cascades but also directly supplies the raw materials for nucleic acid synthesis in regenerating cells. This combination of signaling and substrate support underlies PDRN’s potency in stimulating the tissue repair in wounded, hypoxic, or ischemic environments for neural, skin, muscle and bone cells (Fig. 2) [23]

### Potential roles and recent advances in oral and maxillofacial tissue regeneration

One of the most promising applications of PDRN in dentistry is its role in osteogenesis and angiogenesis. PDRN has been shown to stimulate the proliferation and differentiation of osteoblasts, partly by increasing the expression of critical bone-forming genes such as *RUNX2*, osteocalcin (*OCN*), and osteopontin (*OPN*) [11]. By promoting a richer blood supply, PDRN ensures oxygen and nutrient delivery to regenerating tissues, which is crucial for oral wound healing and bone regeneration [24–26]. Activation of A_2A_ receptors by PDRN markedly increases the expression of VEGF and other angiogenic factors (e.g., angiopoietin-1, MMP-2), thereby facilitating neovascularization in healing bone. Angiogenesis plays a pivotal role in early bone formation, coupling with osteogenesis to ensure successful tissue regeneration. In models of impaired healing such as diabetic wounds and ischemic flaps, PDRN significantly improved angiogenic markers (e.g., CD31, nitric oxide, angiopoietin levels) and accelerated wound closure [27, 28]. These findings underscore the importance of PDRN’s pro-angiogenic effect in supporting tissue repair.

PDRN also upregulates key osteogenic markers at the molecular level. *RUNX2* is a crucial transcription factor in osteoblast differentiation [29]. Meanwhile, collagen type I alpha 1 (*COL1A1*) is an early marker of osteoblast differentiation; it is one of the first genes expressed when progenitor cells commit to the osteogenic lineage, making it a key indicator of the initial stages of bone formation [30]. Although *RUNX2* and *COL1A1* mRNA expression levels varied depending on the concentration of PDRN, the treatment generally influenced gene expression profiles associated with osteogenesis [31]. In vitro, mineralization and calcium deposition were enhanced in gingiva-derived stem cell cultures treated with PDRN, suggesting promotion of the later stages of bone formation [31]. Additionally, PDRN also promoted the growth of cultured human osteoblasts, accompanied by an increase in alkaline phosphatase activity, a marker of osteoblastic activity [24]. Through its pro-angiogenic and pro-osteogenic actions, PDRN creates a synergistic healing environment: new blood vessels support the maturing bone, and the forming bone secretes cytokines that further stabilize the vasculature. This combined action is especially beneficial in dental regenerative procedures (e.g., bone grafts, inflammatory diseases) where both revascularization and new bone formation are critical for success.

#### Bone graft

Considering PDRN’s synergy in promoting angiogenesis and osteogenesis, it can be significant potential for use in dental bone grafting procedures. Yun et al. introduced the concept of osteoimmunology, emphasizing that PDRN exerts its regenerative effects primarily through immunomodulation rather than direct osteogenic stimulation [32]. Specifically, PDRN activates the A2A adenosine receptor pathway, inducing a phenotypic shift of macrophages from the pro-inflammatory M1 type to the anti-inflammatory M2 type. This transition enhances the secretion of IL-10 and VEGF, suppresses TNF-α and other inflammatory cytokines, and establishes an immune environment conducive to angiogenesis, osteoblast activation, and bone remodeling. In 2021, Lim et al. reported in a New Zealand white rabbit model that applying PDRN at concentrations of ≥ 5 mg/mL on a block-type alloplastic scaffold resulted in significantly greater new bone formation at 8 weeks, comparable to the outcome achieved with 0.05 mg/mL of rhBMP-2 [13, 33]. To effectively utilize such hydrophilic agents, however, an appropriate scaffold capable of controlling the release profile in vivo is necessary [34–36]. Collagen sponge has received U.S. FDA approval as a carrier for rhBMP-2. More recently, demineralized dentin matrix (DDM), a type I collagen-based scaffold, has been highlighted as a bioactive carrier [35]. In 2016, PDRN successfully induced bone regeneration when combined with a DDM scaffold, suggesting its potential as a carrier system for clinical use [37]. The present review, however, limits its scope strictly to PDRN-related applications. Beyond preclinical feasibility, recent in vivo studies further support PDRN’s benefits in bone regeneration. Ko et al. conducted an in vivo study in beagle dogs undergoing alveolar ridge preservation procedures, demonstrating significantly higher bone volume and mineral density in PDRN-treated groups compared to controls [38]. Similarly, Lim et al. used a rabbit model of maxillary sinus floor elevation and observed enhanced new bone formation and greater vascularization in the PDRN group at both 2-week and 4-week intervals [39]. Furthermore, histologic analysis revealed that lateral sinus floor elevation augmented with PDRN led to earlier new bone formation and a higher bone-to-implant contact ratio, compared to conventional grafting alone [32, 40]. Although research is still limited on whether PDRN can exhibit osteoinductivity comparable to rhBMP-2 in clinical settings, its angiogenic, anti-inflammatory, and stem cell–stimulating properties are expected to be advantageous.

#### Temporomandibular joint disorder (TMD)

It has been shown that PDRN can help prevent or attenuate numerous inflammation-related conditions, including bursitis of the shoulder and knee joints, fasciitis, and tendinitis [41–46]. PDRN may promote angiogenesis and tissue healing in osteoarthritic joints by down-regulating catabolic and pro-inflammatory mediators (such as tumor necrosis factor-α [TNF-α], interleukin-6 [IL-6], and high mobility group box 1 [HMGB1]) and up-regulating anabolic processes, as demonstrated in an in vitro model of osteoarthritis [47]. PDRN has been observed to modulate key inflammatory cytokines, including TNF-α, IL-1, IL-6, HMGB1, and IL-10, suggesting a potential regulatory role in controlling inflammatory processes. Notably, PDRN exerts its regenerative effects through the activation of adenosine A_2_A receptors, which modulates key inflammatory cytokines (TNF-α, IL-1, IL-6, HMGB1) and elevates anti-inflammatory cytokine IL-10, suggesting a broad regulatory role in inflammation. PDRN’s regenerative effects in musculoskeletal tissues are largely mediated through A_2_A receptor (ADORA2A) activation, which plays a critical role in cartilage and bone regeneration [42]. In vitro, PDRN has shown chondroprotective effects on cartilage cells, and in animal models of arthritis it significantly attenuated joint degeneration and inflammation compared to controls [48–50]. While in vitro studies have demonstrated chondroprotective effects of PDRN and animal models have shown significant attenuation of arthritis compared to controls, clinical studies on temporomandibular joint (TMJ) applications remain scarce. In 2024, Cenzato et al. reported 60 patients with TMJ osteoarthritis and conclude that pericapsular injection of PN and hyaluronic acid effectively reduces pain and improves mandibular kinematics [51]. Although PN and PDRN share structural similarities, their pharmacological properties are distinct. Because this review specifically focuses on PDRN, references to PN have been minimized to maintain clarity and consistency. Recently, prolotherapy using a combination of PDRN and hypertonic dextrose (10% d-glucose) has been reported as a beneficial treatment in TMD patients with TMJ osteoarthritis-related degenerative changes [52]. This regenerative injection approach aims to stimulate healing in ligaments and cartilage, and PDRN’s inclusion adds angiogenic and anti-inflammatory advantages. Additionally, PDRN has been shown to increase the proliferation of human preadipocytes, which could serve as a source of adult stem cells for tissue repair and regeneration [53]. These preadipocytes, when stimulated, have the potential to differentiate and contribute to the repair of musculoskeletal tissues, further supporting the therapeutic rationale for PDRN in TMD and other joint disorders. Recently, Choi et al. analyzed 66 patients with TMD who received prolotherapy with either hypertonic dextrose or PDRN [54]. They found that prolotherapy significantly improved pain and mandibular function, with the mean VAS score dropping from 4.34 ± 2.12 to 1.00 ± 1.58 and MMO increasing from 31.0 ± 8.7 mm to 40.8 ± 4.55 mm (*p* < 0.001). Both treatment groups showed comparable improvements, and 23 patients experienced complete resolution of joint noises, along with significant reductions in mandibular deviation. The authors concluded that prolotherapy—whether using PDRN or dextrose—is an effective intervention for improving pain and jaw function in refractory TMD patients. Similarly, Jang et al. evaluated 111 patients with TMJ osteoarthritis treated with prolotherapy on pericapsular space PDRN injections. They reported a significant reduction in pain (NRS 4.9 to 1.8) and an increase in mouth opening (32.6 ± 8.3 mm to 41.2 ± 5.7 mm; *p* < 0.001) [55]. Notably, 77.5% of patients showed clinically meaningful improvement on the PGIC scale, with greater benefits in acute cases and no adverse effects. Although PDRN demonstrates strong potential as a regenerative therapeutic agent in TMD and TMJ osteoarthritis, the optimal injection protocol—regarding dosage, frequency, and delivery site—has yet to be standardized. Further well-designed randomized controlled trials are warranted to establish the most effective treatment parameters and to validate the long-term safety and efficacy of PDRN-based prolotherapy.

#### Neurological symptoms

Neuropathic pain, resulting from nerve injury or dysfunction of the somatosensory system, can severely diminish a patient’s quality of life because even innocuous stimuli (such as the touch of clothing) may cause persistent pain and discomfort. Various inflammatory substances have been found to induce astrocyte activation, which in turn promotes the development of allodynia, pain from normally non-painful stimuli [56, 57]. Considering PDRN’s anti-inflammatory actions, its potential to ameliorate neuropathic pain has been investigated. Lee et al. used a rat model of induced neuropathic pain to examine whether PDRN could reduce allodynia through anti-inflammatory effects [41, 58]. They found that local PDRN injections at the injury site significantly alleviated mechanical allodynia and decreased the expression of glial fibrillary acidic protein–a marker of astrocyte activation associated with neuroinflammation—in both a spinal nerve ligation model and a chronic post-ischemia pain model. This suggests that PDRN can mitigate neuroinflammatory responses and neuropathic pain symptoms following nerve injury.

Complex regional pain syndrome (CRPS), which can occur after peripheral nerve trauma, is characterized by severe burning pain, allodynia, and hyperalgesia, with symptom severity partly depending on the degree of sympathetic nervous system involvement [59]. Management of CRPS remains challenging, and many patients experience only partial or minimal symptom relief from current treatments. However, PDRN’s combined anti-inflammatory and angiogenic effects may offer a new therapeutic avenue. Activation of A_2__A_ receptors by PDRN leads to increased VEGF levels and enhanced endothelial cell migration and proliferation, improving tissue perfusion and healing. In 2016, the mechanisms of PDRN injection were highlighted as potential benefits for CRPS management, which could help alleviate symptoms by reducing inflammation and improving blood flow in affected tissues [60]. Thus, PDRN might serve as an adjunct or alternative in the treatment of CRPS, although clinical evidence in this specific context is still needed.

Traumatic neuropathies, nerve injuries caused by trauma or surgical procedures, do have an inherent capacity for regeneration, but recovery is often slow and incomplete. To improve recovery from peripheral nerve injuries caused by trauma or surgery, PDRN acts as a non-antigenic, non-toxic agent that strongly stimulates cell proliferation and wound healing, while also promoting angiogenesis [18, 61, 62]. It promotes Schwann cell and fibroblast proliferation, enhances wound healing, and stimulates angiogenesis in the injured nerve’s microenvironment. Improved blood supply is essential for delivering oxygen and nutrients to regenerating nerves, and PDRN’s pro-angiogenic effect likely contributes to a more conducive environment for nerve regrowth. Inflammatory cytokines such as IL-1 and IL-6, which can exacerbate nerve injury and edema, were found to be downregulated by PDRN treatment, leading to reduced swelling and secondary damage in injured nerves [63]. Studies have shown that PDRN treatment can accelerate neural tissue repair and functional recovery. Moreover, in a mouse sciatic nerve transection model, PDRN combined with VEGF (which PDRN is known to upregulate) produced superior regenerative effects [64]. In 2025, Sun et al. reported that PDRN and low-level laser therapy (LLLT) acted synergistically to enhance peripheral nerve regeneration in a crush-injured facial nerve model [65]. These findings suggest that PDRN could be a promising therapeutic for accelerating nerve regeneration and treating neuropathic pain following craniofacial nerve injuries—such as trauma to the inferior alveolar nerve—PDRN could be considered a promising therapeutic option to promote nerve regeneration and to manage neuropathic pain.

#### Inflammatory oral disease

Most inflammatory conditions within the oral cavity originate from bacterial endotoxins released by biofilms. Biofilms accumulating on non-shedding surfaces—such as teeth, bone, and dental implants—require mechanical removal, as they are resistant to host immune responses and pharmacologic interventions [66, 67]. Peri-implantitis including periodontitis and medication-related osteonecrosis of the jaw (MRONJ) are two challenging oral conditions characterized by inflammation-driven bone loss and impaired healing. Contemporary treatment guidelines emphasize the effective decontamination and removal of these pathogenic biofilms and associated inflammatory and necrotic tissues.

MRONJ is also believed to originate from a state of drug-induced ischemia followed by antiresorptive or antiangiogenic agents [68]. Recent in vitro studies have demonstrated the potential of PDRN in mitigating such effects. PDRN has shown cytoprotective effects in macrophage cultures treated with zoledronic acid and lipopolysaccharide (LPS), where the combination of bacterial endotoxin and bisphosphonate synergistically reduced cell viability and increased inflammatory cytokine expression [22]. Notably, supplementation with PDRN significantly improved cell survival and downregulated inflammatory mediators. Jung et al. proposed that PDRN could serve as a valuable adjunct in MRONJ management by accelerating soft tissue healing and reducing the recurrence of osteonecrotic lesions [69]. Infection and inflammation have long been considered critical cofactors in the pathogenesis of osteonecrosis of the jaw (ONJ). Even when necrotic bone is exposed, lesions may remain asymptomatic if bacterial invasion does not occur. Conversely, inflammation and recurrence of MRONJ often follow surgical debridement if residual bone remains colonized by pathogenic bacteria [68, 70]. Hence, effective control of local biofilms has emerged as a crucial element in MRONJ therapy. Biofluorescence imaging devices have recently been introduced to detect and visualize bacterial biofilms intraoperatively, allowing for more accurate and complete debridement of necrotic tissues [70–72]. However, once inflammation has led to structural degradation of surrounding soft or hard tissue, merely removing the biofilm does not guarantee functional tissue restoration. It is well recognized that the healing capacity of the lesion is compromised if the surrounding supporting tissues have been destroyed [66]. In this context, PDRN—through its dual mechanism involving nucleotide salvage and adenosine A_2__A_ receptor activation—may suppress persistent inflammation while promoting tissue regeneration, thus reducing the risk of recurrence after treatment. Some studies support that PDRN’s ability to prevent osteonecrosis induced by both chemical (e.g., intra-articular monoiodoacetate injection) and mechanical insults (e.g., anterior cruciate ligament injury), as well as bisphosphonate exposure [73, 74].

These findings support the use of PDRN as a therapeutic adjunct in early-stage peri-implant mucositis and in “stage 0” MRONJ—that is, in patients who are at risk but have not yet developed clinically exposed necrotic bone. Particularly noteworthy is PDRN’s potent pro-angiogenic activity, which may be advantageous in disorders characterized by avascular necrosis (e.g., MRONJ) as well as in peri-implant bone sclerosis driven by chronic inflammation [11, 12, 14, 15, 71, 75]. Accumulating in-vitro and preclinical evidence indicates that PDRN helps preserve cell viability, attenuate inflammatory cascades, and protect bone homeostasis, thereby reinforcing its therapeutic promise for peri-implantitis [75, 76]. In 2025, systematic review concluded that PDRN significantly enhances periodontal regeneration by stimulating bone and soft-tissue healing while concurrently dampening local inflammatory responses [12]. In such clinical scenarios, the compound’s combined anti-inflammatory, angiogenic, and regenerative actions offer a biologically active, minimally invasive strategy to halt disease progression and promote functional tissue recovery. In 2025, guided tissue regeneration in an acute periodontal abscess using PDRN pre-soaked with cross-linked collagen matrix, along with submucosal PDRN injections intraoperatively and two weeks postoperatively, showed stable soft tissue architecture, a reduced probing depth (2 mm), and no evidence of bone loss progression [77]. However, clinical studies on the treatment of such inflammatory lesions are still lacking.

#### Oral mucosa autoimmune diseases

As an immunomodulatory agent with tissue-healing properties, PDRN has been explored in the management of oral mucosal autoimmune conditions [78, 79]. One example is oral lichen planus (OLP), a chronic inflammatory disease whose pathogenesis involves a T-cell mediated, epithelium-directed inflammation in response to unknown antigen that affects the oral mucosa [80]. Laino et al. reported that intralesional PDRN injections provided adjuvant therapeutic effects in patients with OLP, helping to reduce lesion size and inflammation (particularly when used alongside conventional treatments) [78]. In a related autoimmune condition, lichen sclerosus (a mucocutaneous disorder, commonly affecting genital skin), PDRN dermal infiltration was shown to have beneficial adjunct effects when combined with topical therapy, suggesting improvements in tissue quality and symptom relief [79]. Another area of investigation is the mitigation of oral mucositis, which often occurs as a complication of radiation therapy in head and neck cancer patients. Topical application of PDRN in a cohort of patients with radiation-induced oral mucositis led to reduced severity of mucosal ulceration and faster healing [81]. Collectively, these studies indicate that PDRN may serve as a useful adjunct in treating oral autoimmune and inflammatory mucosal diseases by promoting tissue repair and modulating local immune responses.

## Future direction and conclusion

**Fig. 3:**
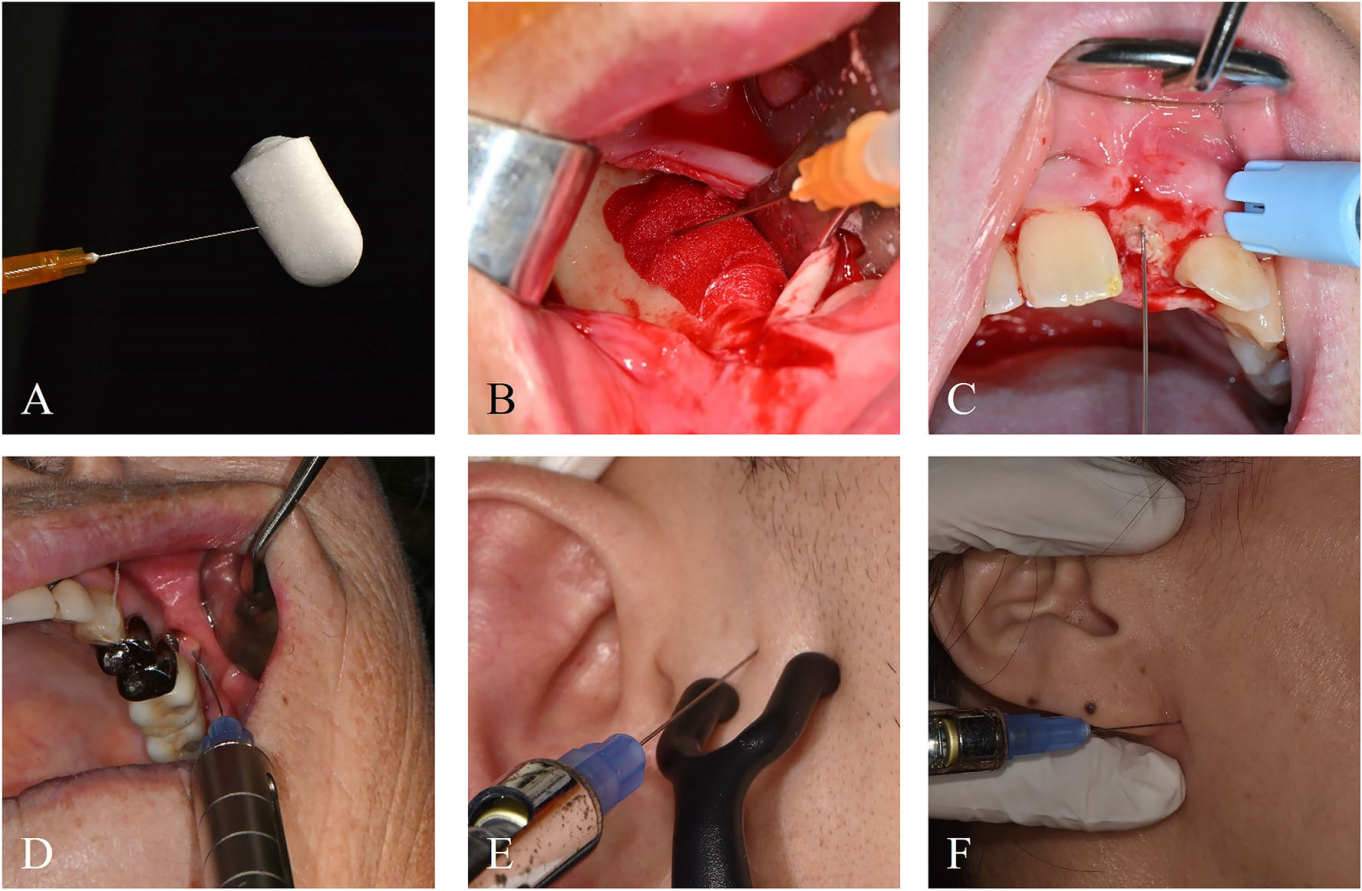
Various clinical applications of PDRN. **A** Soaking of collagen sponge as a carrier. **B** Injection into bone defect after surgical debridement of MRONJ. **C** Injection at the dehiscence area showing insufficient gingival healing after bone graft surgery. **D** Injection with a digital automatic painless injector (i-JECT, Medihub, Gunpo-Si, Republic of Korea) around peri-implantitis and mucositis sites. **E** Intracapsular injection into the TMJ. **F** Pericapsular prolotherapy for TMD.

From the evidence reviewed, PDRN emerges as a promising biologically active compound in oral and maxillofacial regenerative therapy. Unlike agents that target single molecular pathways, PDRN activates the adenosine A_2_A receptor and simultaneously provides nucleotide precursors through the salvage pathway. This dual mechanism facilitates a broad range of cellular responses, including inflammation control, angiogenesis, and tissue repair. Its demonstrated efficacy across periodontal healing, bone regeneration, TMJ disorders, and intraoral autoimmune and inflammatory conditions reflects its translational potential in dentistry. (Table 1, Fig. 3)

**Table 1:** Summary of Preclinical and Clinical Research of PDRN in Dentistry

Model/Design	Application	Key Findings	Ref. [75]
Rat (in vivo)	Experimental periodontitis	Arrested inflammatory response (MAPKs (p-JNK and p-ERK), pro-inflammatory cytokines (TNF-α, IL-6, HMGB-1) and the pro-apoptotic BAX) and preservation of the gingivo-mucosal layer	
Nude mice (in vivo)	Osteoinductivity with demineralized dentin matrix (DDM)	Induced bone regeneration when combined to the DDM	[37]
Hubman oral mucosal epithelial cells and gingival fibroblasts (in vitro)	Oral mucositis	Blunted inflammation and improved wound healing	[85]
New Zealand white rabbit (in vivo)	Calvaria defect	Increased alkaline phosphatase, osteoblast differentiation and new bone formation	[13]
Human periodontal ligament (PDL) fibroblasts (in vitro)	PDL cells store	Exhibited cell-preserving and anti-inflammatory effects on the PDL cells.	[76]
Canine (in vivo)	Sinus augmentation + implant placement	Early new bone formation and higher bone-to-implant contact (BIC)	[40]
Canine (in vivo)	Sinus augmentation + implant placement	Greater BIC and trabecular thickness	[11]
Beagle dog (in vivo)	Alveolar ridge preservation	Increased bone volume and mineral density	[38]
Gingival stem cell culture (in vitro)	Osteogenic differentiation	Increased ALP, collagen, and mineralization	[31]
Canine (in vivo)	Buccal peri-implant bone defect repair	Accelerated bone healing using collagen matrix + PDRN	[15]
Rabbit (in vivo)	Maxillary sinus floor elevation	Enhanced bone formation and vascularization at 2 and 4 weeks	[39]
Radiation-induced Oral mucositis (n = 3)	Mucosal spray	Topical PDRN spray reduced mucositis grade (G3→G1–2) within one week; rapid pain relief; safe with no allergic reactions.	[81]
Post-MRONJ surgical defect healing (n = 5)	Mucosal injection	Soft tissue regeneration, preliminary but promising	[69]
TMJ DJD (case series, n = 4)	TMJ prolotherapy	Improved VAS pain, MMO, joint noise, and condylar surface contour	[52]
Acute periodontal abscess (case report, n = 1)	Guided Tissue regeneration, Mucosal injection	Successful infection control and guided tissue healing after PDRN application.	[77]
TMJ-OA (Acute vs. Chronic TMJ-OA patient, n = 111)	TMJ prolotherapy on pericapsular space	Significant improvement in NRS pain and MMO; 77.5% patients’ subjective perception of treatment efficacy response; greater effect in acute (<3 months) than chronic cases.	[55]
TMD (PDRN Vs. Hypertonic dextrose, n = 66)	TMJ prolotherapy on posterior disc attachment tissues, anterior disc attachment tissues, the superior portion of the lateral capsule, the inferior portion of the lateral capsule	Similar to hypertonic dextrose, Comparable clinical improvement in pain and MMO; PDRN showed earlier symptomatic relief.	[54]

Additionally, PDRN exhibits favorable pharmacokinetics with a peak concentration approximately 1 hour after intramuscular administration and a half-life of 3.5 hours, and a bioavailability ranging from 80 to 90% [1]. Toxicological evaluations confirmed that repeated systemic administration of PDRN at doses up to 8 mg/kg did not result in mortality or adverse macroscopic findings in the liver, lungs, brain, skeletal muscle, or heart [82–84]. The broad therapeutic profile, along with its high safety margin and regenerative efficacy, underscores PDRN’s potential as a minimally invasive adjunct to enhance current regenerative protocols in dental practice and significant potential for broader indications in dentistry including bone regeneration, periodontal repair, implant therapy, MRONJ management, and neurovascular healing. However, current evidence is largely derived from preclinical studies and small-scale clinical reports. Future clinical studies, particularly well-designed clinical trials, will be essential to validate its long-term efficacy and define its precise role within evidence-based treatment protocols and optimal delivery systems to maximize therapeutic outcomes in diverse oral diseases.

## Data Availability

The datasets generated during and/or analysed during the current study are available from the corresponding author on reasonable request.
